# Depression and Acute Myocardial Infarction: What’s New in Their Association?

**DOI:** 10.1192/j.eurpsy.2025.1352

**Published:** 2025-08-26

**Authors:** K. M. Panoutsopoulou, M. Efstathiou, A. Grammeniati, S. Mantzoukas, M. Gouva, K. K. Naka, E. Dragioti

**Affiliations:** 1 Department of Nursing, Research Laboratory Psychology of Patients, Families & Health Professionals; 2 Department of Nursing, Research Laboratory Intergrated Care, Health & Well-being; 3 Department of Medicine, Second Department of Cardiology, University of Ioannina, School of Health Sciences, Ioannina, Greece

## Abstract

**Introduction:**

Depression and myocardial infarction (MI) are leading causes of disability worldwide. Their interconnectedness, along with the burden that these impose on patients, is of particular clinical interest.

**Objectives:**

We conducted a systematic review, that aimed to synthesize recent data on the association between depression and MI, highlighting their outcomes and identifying contributing risk factors.

**Methods:**

Data were extracted from 17 studies, including only prospective and longitudinal cohort studies, published between 2019 and 2024. These studies were selected from an initial pool of 67 relevant articles, retrieved from PubMed, PsycINFO, and Embase databases.

**Results:**

Prevalence of depression was higher among MI survivors (OR 1.21; 95% CI 1.15-1.27), with increased rates observed in patients with comorbid type 2 diabetes (IR 131.1; 95% CI 109.6-155.6). A significant correlation was found between depression and adverse clinical outcomes post-MI (HR 3.41; 95% CI 2.49-2.674), including 30-day hospital readmissions (HR 1.11; 95% CI 1.07-1.15). Additionally, individuals diagnosed with depression had a higher likelihood of experiencing MI (HR 2.07; 95% CI 1.79-2.40), particularly those from lower socioeconomic backgrounds (HR 1.47; 95% CI 1.36-1.60) or with chronic kidney disease (HR 1.29; 95% CI 1.03-1.62). However, depressed men and patients using SSRIs had a reduced risk of MI compared to women and non-SSRI users (HR 1.39; 95% CI 1.35-1.42 and HR 0.91; 95% CI 0.64-1.29, respectively).

**Conclusions:**

Depression increases the risk of developing MI and worsens clinical outcomes, and vice versa. Comorbid conditions, including type 2 diabetes and chronic kidney disease, gender differences, and no antidepressant use, are critical factors that compound their association.

**Disclosure of Interest:**

None Declared

